# Short-term intracavernous self-injection treatment of psychogenic erectile dysfunction secondary to sexual performance anxiety in unconsummated marriages

**DOI:** 10.1038/s41443-020-00399-z

**Published:** 2021-02-18

**Authors:** Ghalib Lidawi, Mohsin Asali, Muhammad Majdoub, Ronen Rub

**Affiliations:** Department of Urology, Hillel Yaffe Medical Center, Hadera, Israel

**Keywords:** Erectile dysfunction, Quality of life

## Introduction

Erectile dysfunction (ED), the inability to achieve and/or maintain an erection sufficient for satisfactory sexual intercourse is traditionally considered an age dependent male dysfunctional disorder, resulting from the physiological changes associated with the aging process [1]. Previous surveys demonstrated an age associated 3-fold increased probability of ED in men between ages 20–60 [2]. However, in recent multinational studies, the prevalence of ED in young men (under 40) has been shown to be as high as 30% [3].

The hemodynamic changes associated with achieving an erection are dependent on the seamless synergistic interplay between healthy psychologic, neurologic, endocrinologic, metabolic, and vascular factors [4]. In younger men without vascular risk factors, psychogenic ED is generally considered to be the main etiology of ED [5, 6]. Psychogenic ED is predominantly or exclusively due to psychologic or interpersonal factors including: sexual performance anxiety, global anxiety, relationship and social factors, depression, guilt and fear [6, 7].

Physicians treating patients with psychogenic ED in certain conservative societies frequently face situations that are uniquely related to the specific culture and family attitudes. Newly married couples in conservative societies may have limited premarital sexual experiences. The men might be under a significant amount of stress, which affects their ability to achieve an erection leading to unconsummated marriage, a condition in which newly married couples are unable to achieve penile-vaginal intercourse for variable periods despite the couple’s desire and numerous attempts. Moreover, when psychogenic ED is left unresolved, fear of failure may preclude future attempts, frequently resulting in a vicious cycle of sexual performance anxiety, often with the cessation of all sexual activity and increased distancing within the relationship [5, 8].

One of the many benefits of intracavernous injection (ICI) therapy, it is a purely physiological treatment that offers an almost instant natural erection; this alone reduces anxiety in patients with a substantial psychogenic component in their ED [9].

In the case series presented herein we assessed the success of at-home use of self-injected ICI treatment following diagnosis of psychogenic ED, secondary to sexual performance anxiety, in young men from traditional Muslim communities who reported on unconsummated marriages.

## Our observation

This case series conducted between 2017 and 2019 evaluated ICI treatment in 12 patients, average age 30 (range 20–45 years), at the Clinic for Sexual Health at Hillel Yaffe, a university affiliated Medical Center in Northern Israel serving a population of half a million residents, 40% of which are Muslim, Christian and Druze Arabs.

The medical and sexual history of all patients was recorded. All young men from traditional Muslim communities reported on unconsummated marriages and sought treatment on an average of 18 months (range 1–3 years) after their wedding. All but one had no sexual experience before marriage. Our cohort of patients were young healthy men; thus, no additional lab or other tests were conducted.

According to the data collected, none of the patients had difficulty achieving an erection prior to penetration. A clear history of full rigid morning or night erections and having a healthy libido was reported in all but 2 patients. One of these patients did not engage in masturbation, thus, all but one patient reached ejaculation. In all 12 men, the etiology for the diagnosis of ED was psychogenic, without vascular risk factors.

All patients were treated for psychogenic ED secondary to sexual performance anxiety. All patients failed to respond to repeated trials on phosphodiesterase type 5 (PDE5) inhibitors. Nine of 12 patients on treatment with PDE5 inhibitors achieved an erection that was lost before penetration. All couples were offered and refused sex therapy. Seven patients were smokers and two of the couples were in consanguineous marriages (related cousins).

### Psychogenic ED secondary to sexual performance anxiety treatment with ICI

Of note, all of the patients participating in our study were highly motivated to succeed. For this study, the treatment procedure was according to the following ICI therapy protocol:

After the initial in-clinic training session with a demonstration of the self-injection technique on a model of a penis and detailed explanations on the sterilization and injection technique, patients were sent home with one 1 ml syringe fitted with “27Gx1/2” needle prepared and ready for use, with the appropriate concentration of low dosage combination of papaverine and alprostadil. According to the ICI protocol starting with 0.5 ml solution that contains 6 mg papaverine and 6 mcg alprostadil. If a satisfactory erection was not achieved, at the second treatment session the dose was titrated to 1 ml. solution of 12 mg of papaverine and 12 mcg of alprostadil. Following self-injection at home, the patients were instructed to wait 15–20 min for response and only then to engage in sexual intercourse.

Treatment and outcomes: Seven of the twelve (58.3%) patients reported achieving good erection after one treatment with ICI self-injected at home that led to successful sexual intercourse with penetration, thus experiencing immediate success.

The remaining 5/12 (41.6%) required one additional ICI treatment after several weeks with a titrated dose, to report good erection and experience successful sexual intercourse with penetration.

No side effects, as priapism, were noted in any of the cases. This can be explained by the low dosages that were used, even when the titration was higher.

Follow-up data was available for all patients. Following 1 to 2 ICI treatments all patients reported on resumption of spontaneous erections and were able to end the cycle of sexual performance anxiety and engage in continued sexual intercourse without the need for further treatment. On follow-up it was observed that 8/12 (75%) of the couples conceived within an average of 8 months (range 2–24 months) and went on to have a normal pregnancy.

### Our perspective

During recent years the Clinic of Sexual Health has focused on treatment of younger men from traditional Muslim communities diagnosed with psychogenic ED, secondary to sexual performance anxiety. All of the men in the current case series had no sexual experience (apart from one previously married) and all reported on unconsummated marriages and sought treatment on an average of 18 months (range 1–3 years) after their wedding. In unresolved psychogenic ED the vicious cycle of sexual performance anxiety frequently results in the loss of sexual or affectionate interaction, which is associated with increased performance demands and interpersonal distress [5, 8].

In these cases of psychogenic ED, following previous treatment failure with PDE5 inhibitors, we offered the couples a simple, inexpensive, short term intervention: a brief course of ICI of vasoactive drugs. Patients reported good erection after one to two treatments with intracavernous self-injection leading to successful sexual intercourse. Due to successful ICI therapy, our patients experienced resumption of spontaneous erections, most probably due to the resolution of performance anxiety [10], therefore they no longer had need for further treatment. Thus, they effectively ended the cycle of sexual performance anxiety, which led in these couples to an improved sexual quality of life. As such, ICI therapy may have, in addition to improving their sexual life, also contributed to their quality of life, possibly even preempting feelings of marriage failure and depression [6].

Societies in which couples have enough opportunity for premarital sex, rarely report on unconsummated marriage. In contrast, in traditional and conservative societies, premarital sex is strictly prohibited and is considered scandalous and an insult to the family honor [11], therefore, the majority of current publications on unconsummated marriage originate from Eastern or Middle Eastern countries as Iran [11, 12], Turkey [13], Tunisia [14], and Egypt [15, 16]. Indeed, in many of these reports, psychogenic ED was considered to be the major etiological factor of unconsummated marriage, secondary to performance anxiety. Previous similar studies with young males from traditional conservative populations diagnosed with ED also recognized secondary sexual performance anxiety, thus chose ICI therapy.

While we demonstrated that a brief course (1-2 self-injections) of ICI was sufficient in all patients to overcome the psychogenic barriers of sexual performance anxiety, other studies with similar populations reported up to 30% required 3 or 4 ICI treatments [11, 14].

One study with a similar in clinic ICI self-injection training and dose titration protocol reported 46 (64%) patients required up to 3 months of weekly self-injections, and 15(21%) continued up to a year [15].

Notably, in our clinic we practice extreme caution in dosing and the chosen protocol used for ICI was with a 1 ml syringe fitted with a small “27Gx1/2” needle to minimize the pain of injection and limit the chance of bleeding. Moreover, treatment was with the lowest effective dosages in order to avoid side effects. Of note, the relatively young age and healthy status of the study group who were also highly motivated toward success from the initial training on self-injection. Indeed, the majority of our ICI therapy patients used 0.5 ml solution of combination therapy 6 mg papaverine and 6 mcg alprostadil and only in 5 patients ICI therapy required titration up to 1 ml solution of 12 mg of papaverine and 12 mcg of alprostadil. Consequently, in our study the use of short-term low dose ICI was also quite safe. There were no events of side effects, as priapism, in the any of the patients.

However, in stark contrast to our ICI self-injected low dose protocol, in other studies, in all cases ICI was injected by the urologist in the clinic (as the majority of patients came from remote rural areas) and therefore the patients were instructed to go home immediately to attempt intercourse. Thus, significant differences were notable between ICI protocols, as the injections had to be titrated to relatively higher dosages so that the patients could reach home and attempt intercourse before detumescence occurred.

Indeed, the reports of higher dosages of intracavernous vasoactive drugs used were of papaverine 20 mg titrated to 40 mg [13] or up to 60 mg papaverine [14] or a mean dose of 70 mg papaverine or combination therapy of 10 mg phentolamine with papaverine 40-mg [13] up to 120 mg[12]. Consequently, due to the higher dosages of intracavernous vasoactive agents used in these studies often high rates of side effects, as prolonged erection and priapism (49%) and pain (47%) were reported [11, 12, 16].

Although the gold standard, treatment of psychogenic ED in sex therapy, is often a long, expensive process, which may result in treatment failure, often due to social and interpersonal factors, as the lack of the couples’ acceptance and compliance [5]. Consequently, very few studies in the literature, among a similar population, reported on compliance with sessions of sex therapy, resulting in various degrees of successful outcomes: from success of concomitant sex therapy with PDE5 inhibitors [13], to partial success in only 10 of 57 couples [14], while others reported all patients began ICI therapy following failed sex therapy [15].

In our experience, the cultural acceptance of joint couple therapy was greatly compromised in our study population leading to refusal in all patients to participate. Similar noncompliance was stated in other studies on couples from traditional societies [11, 12]. Indeed, the Iranian urologist who conducted these two large studies claims ICI should be regarded as first-line treatment in cases of unconsummated marriages, instead of prolonged, expensive psychotherapy, since deep, complex psychological problems were most probably not a major cause in the vast majority of cases. Moreover, ICI creates a very rapid response and had shown a curative effect in the majority, without any need for maintenance therapy [11, 12].

### Limitations

The small size of our study group is the main limitation. Although, good success rates were noted in our trial, further follow-up is needed to determine the recurrence rate of the problem and if a brief course of ICI is sufficient to overcome the psychogenic barriers.

## Conclusion

In these cases of unconsummated marriages due to psychogenic ED, secondary to sexual performance anxiety in young healthy men, we found that short-term treatment (1–2 self-injections) with low dose intracavernous pharmacotherapy was a safe, simple, inexpensive and effective alternative in cases of previous treatment failure with PDE5 inhibitors. Moreover, due to successful ICI therapy the patients experienced resumption of spontaneous erections, with the resolution of the cycle of sexual performance anxiety. In addition to improving their sexual life, it may also have contributed to their quality of life, possibly even preempting feelings of marriage failure and depression.

## References

[1] National Institutes of Health. Consensus development conference statement on impotence. Int J Impot Res. 1993;5:181–284. 8173631

[2] Kubin M, Wagner G, Fugl-Meyer AR (2003). Epidemiology of erectile dysfunction. Int J Impot Res.

[3] Nguyen HMT, Gabrielson AT, Hellstrom WJG (2017). Erectile dysfunction in young men-a review of the prevalence and risk factors. Sex Med Rev.

[4] Leung AC, Christ GJ, Melman A Physiology of penile erection and pathophysiology of erectile dysfunction. In: Lue TF (ed.). Atlas of male sexual dysfunction. Current Medicine Group: London, 2004, pp 1–25.

[5] Rosen RC (2001). Psychogenic erectile dysfunction. Classification and management. Urol Clin North Am.

[6] Bodie JA, Beeman WW, Monga M (2003). Psychogenic erectile dysfunction. Int J Psychiatry Med.

[7] McMahon CG (2019). Current diagnosis and management of erectile dysfunction. Med J Aust.

[8] Yafi FA, Jenkins L, Albersen M, Corona G, Isidori AM, Goldfarb S (2016). Erectile dysfunction. Nat Rev Dis Prim.

[9] Lue TF Evaluation and nonsurgical management of erectile dysfunction and priapism. in Walsh PC, Retik AB, Vaughn ED, Wein AJ, eds. *Campbell’s urology* Philadelphia: Saunders 1998;1181–214.

[10] Kandeel FR, Koussa VK, Swerdloff RS (2001). Male sexual function and its disorders: physiology, pathophysiology, clinical investigation, and treatment. Endocr Rev.

[11] Zargooshi J (2000). Unconsummated marriage: clarification of aetiology; treatment with intracorporeal injection. BJU Int.

[12] Zargooshi J (2008). Male sexual dysfunction in unconsummated marriage: long-term outcome in 417 patients. J Sex Med.

[13] Shamloul R (2006). Management of honeymoon impotence. J Sex Med.

[14] Nabil Mhiri M, Smaoui W, Bouassida M, Chabchoub K, Masmoudi J, Hadjslimen M (2013). Unconsummated marriage in the Arab Islamic world: Tunisian experience. Sexologies.

[15] Ghanem H, Sherif T, Adbel-Gawad T, Asaad T (1998). Short term use of intracavernous vasoactive drugs in the treatment of persistent psychogenic erectile dysfunction. Int J Impot Res.

[16] Badran W, Moamen N, Fahmy I, El-Karaksy A, Abdel-Nasser TM, Ghanem H (2006). Etiological factors of unconsummated marriage. Int J Impot Res.

